# Intratumoral expression of FoxP3-positive regulatory T-cells in T-cell lymphoma: no correlation with survival

**DOI:** 10.1080/03009734.2018.1555195

**Published:** 2019-03-11

**Authors:** Josefine Lundberg, David Berglund, Daniel Molin, Amelie Kinch

**Affiliations:** a Department of Immunology, Genetics and Pathology, Section of Clinical Immunology, Uppsala University, Uppsala, Sweden;; b Department of Immunology, Genetics and Pathology, Section of Experimental and Clinical Oncology, Uppsala University, Uppsala, Sweden;; c Department of Medical Sciences, Section of Infectious Diseases, Uppsala University, Uppsala, Sweden

**Keywords:** FoxP3, outcome, T-cell lymphoma, Tregs, tumor microenvironment

## Abstract

*Background*. In cancer, regulatory T-cells (Tregs) were previously believed to inhibit tumor immunity, leading to reduced survival. However, in hematologic malignancies, including T-cell lymphoma (TCL), a correlation between increased numbers of tumor-infiltrating Tregs and a favorable prognosis has been reported. We aimed to investigate the expression of the Treg biomarker forkhead box protein 3 (FoxP3) in TCL in immunocompetent individuals and explore a possible correlation to overall survival.

*Methods*. In total, 35 diagnostic biopsies of TCL were stained using a FoxP3-specific monoclonal antibody (clone 236A/E7). Visual scoring was performed by counting positive cells in 15 high-power fields. Clinical data were collected retrospectively from medical records.

*Results*. All the TCLs contained FoxP3^+^ cells, median 342 FoxP3^+^ cells/mm^2^ (range 1–3047). The degree of intratumoral expression of FoxP3 varied between the different subtypes of TCL, with the highest frequency found in angioimmunoblastic TCL. The frequency of intratumoral FoxP3^+^ cells had no impact on overall survival; neither when using a cutoff value of 200 FoxP3^+^ cells/mm^2^ (*P* = 0.84) nor with FoxP3 as a continuous variable (*P* = 0.63).

*Conclusions*. Intratumoral Tregs are frequently found in TCL in immunocompetent individuals. In this heterogeneous group of TCL, there was no correlation between the density of intratumoral FoxP3^+^ cells and overall survival.

## Introduction

Regulatory T-cells (Tregs) are a specialized subpopulation of T-cells with the capacity to suppress anti-self immune responses, thus maintaining peripheral tolerance and preventing autoimmune diseases (1). Tregs are also believed to play a vital part in inducing tumor-specific immune tolerance (2). Natural Tregs are produced in the thymus (3) and express CD4, CD25, and the transcription factor forkhead box protein 3 (FoxP3) (4). Peripheral CD4^+^ FoxP3^−^ cells may also upregulate FoxP3 in the presence of cytokines such as TGF-β and IL-2; this population of extrathymically generated FoxP3^+^ T-cells are known as peripherally derived Tregs (pTregs) (5). The transcription factor Helios (6) and the cell surface molecule neuropilin-1 (7) have been suggested as markers to distinguish nTregs from pTregs, but the usefulness of these markers in humans has been subject to debate (8,9), and consensus has not yet been reached. Despite the fact that CD4^+^ CD25^−^ T-cells have been shown to express FoxP3 upon activation, and that the levels of expression do not necessarily correlate to suppressive function in these cells, FoxP3 is still considered the most reliable molecular marker of Tregs (4, 10).

Regarding the role of Tregs in cancer, several studies have shown a correlation between increased numbers of intratumoral Tregs and decreased survival (e.g. ovarian carcinoma (11), prostate cancer (12), and non-small cell lung cancer (13)). In contrast, a higher frequency of intratumoral Tregs has been associated with improved survival in colorectal carcinoma (14) as well as in several types of lymphoma—for example, follicular lymphoma, Hodgkin lymphoma, and germinal center-like diffuse large B-cell lymphoma (15). In previous studies of Tregs in T-cell lymphoma (TCL), a positive impact on survival with increased numbers of intratumoral Tregs has been reported in mycosis fungoides (MF), unspecified cutaneous TCL (CTCL) (16), and extranodal NK/T-cell lymphoma (ENKTL) (17). One study compared lymph node biopsies from patients with angioimmunoblastic TCL (AITL) or follicular lymphoma with reactive lymph nodes and found significantly lower numbers of Tregs in the AITL biopsies, which, according to the authors, might at least in part explain the autoimmune symptoms typical for AITL and thus the poor prognosis (18). Another study on adult T-cell leukemia/lymphoma (ATLL) focused on the expression of FoxP3 in the malignant cell population and reported that patients with FoxP3-negative tumors showed a tendency toward better survival in comparison with those with strong or intermediate intratumoral expression of FoxP3 (19).

In a previous study we found that none of the T-cell posttransplant lymphoproliferative disorders were FoxP3^+^ (20). We speculated that this finding in part might be due to the immunosuppressive treatment that the patients typically receive after solid organ transplantation, and we questioned if TCL from patients without any previous immunosuppression would differ in their expression of FoxP3, which led us to initiate this present study. We hypothesized that increased numbers of intratumoral Tregs may have an impact on survival in different types of TCL, and in this study we have correlated the number of FoxP3^+^ cells in biopsies of different subtypes of TCL to outcome.

## Materials and methods

We included 35 TCL patients diagnosed 1999–2002 with sufficient tissue available for further analysis from a Swedish–Danish case-control study (SCALE; Scandinavian Lymphoma Etiology) (21). The 35 tissue samples were re-evaluated in the SCALE study by experienced hematopathologists according to the World Health Organization (WHO) classification of tumors in hematopoietic and lymphatic tissue. After re-examination of the pathological reports in combination with the clinical data, we reclassified one of the biopsies from primary cutaneous CD30^+^ TCL to TCL not otherwise specified. Another biopsy was reclassified as highly malignant TCL not otherwise specified, after consulting an experienced hematopathologist, since the original diagnosis of ‘peripheral large cell TCL’ was not consistent with any of the diagnostic entities of the WHO classification. Clinical data for each patient were collected retrospectively from the different Swedish hospitals where the patients had been primarily treated for their TCL.

### Immunohistochemistry for FoxP3

From formalin-fixed, paraffin-embedded diagnostic biopsies of TCL, 4 μm thick tissue sections were sliced. PT-link was used for performing deparaffinization and antigen retrieval. Immunohistochemical (IHC) staining was performed with Dako autostainer plus with clone 236A/E7 (eBioscience) used as primary antibody to detect FoxP3, diluted 1:100 in antibody-solution (Dako). In order to visualize the primary antibody, EnVision DAB-kit (Dako) was used. The samples were scanned and examined using Aperio ImageScope v12.1.0.5029 (Leica Biosystems). In the majority of the samples the positive cells were distributed in a heterogeneous manner, therefore the stained cells were counted manually in 15 arbitrarily chosen fields in each biopsy, at 40× magnification. The area of each field was measured. The number of positive cells per mm^2^ in the total analyzed area was calculated.

### Statistics

Comparison of frequencies of FoxP3^+^ cells in different subtypes of TCL was made using the Mann–Whitney *U*-test. Overall survival (OS) was defined as the time from initial diagnosis of TCL until death or last date of follow-up, which was 19 February 2016. Survival curves were generated using the Kaplan–Meier method. Difference in survival was calculated using Cox proportional hazards regression model with FoxP3 as a continuous variable or log-rank test with a cutoff value of 200 FoxP3^+^ cells/mm^2^. Statistical significance was defined as *P* < 0.05. All statistical analyses were performed using Statistica software (Version 13, Stat Soft Inc., Tulsa, OK, USA).

#### Ethics approval

The study was approved by the Regional Ethical Review Board in Stockholm, Sweden, and was conducted in accordance with the Declaration of Helsinki.

## Results

### Clinical characteristics and subtypes of TCL

The median age at the time of diagnosis was 57 years (range 18–73) (Table 1). There were 21 men and 14 women. The majority of the patients (57%) had extranodal localization of TCL, most commonly the skin (20%). The most common subtype of TCL was anaplastic large cell lymphoma (ALCL; *n* = 12, 34%). Other subtypes included peripheral TCL, not otherwise specified (PTCL-NOS; *n* = 6, 17%), unspecified TCL (*n* = 5, 14%), AITL (*n* = 3, 9%), enteropathy-associated TCL (EATL; *n* = 3, 9%), CTCL (*n* = 2, 6%), MF (*n* = 2, 6%), and T-cell prolymphocytic leukemia (T-PLL, *n* = 2, 6%).

**Table 1.** Clinical and biological characteristics of the patient group.

Characteristics	Number (%) (Available data)
Sex	(35/35)
Male	21 (60%)
Female	14 (40%)
Age at TCL diagnosis	(35/35)
18–20	2 (6%)
41–50	8 (23%)
51–60	13 (37%)
61–70	11 (31%)
>70	1 (3%)
TCL localization	(35/35)
Nodal^a^	15 (43%)
Extranodal	20 (57%)
Bone marrow	6 (17%)
Skin	7 (20%)
Small intestine	5 (14%)
Other^b^	6 (17%)
Stage (Ann Arbor)	(31/35)
I	9 (29%)
II	4 (13%)
III	11 (35%)
IV	7 (23%)
Performance status (WHO/ECOG)	(35/35)
0–1	33 (94%)
2–3	2 (6%)
IPI	(26/35)
Low risk	11 (42%)
Low/high intermediate risk	14 (54%)
High risk	1 (4%)
Serum LDH concentration	(28/35)
Elevated	15 (54%)
Within normal range	13 (46%)
Recurrence	(35/35)
Yes	19 (54%)
No	16 (46%)
Subtypes of TCL	(35/35)
Anaplastic large cell lymphoma	12 (34%)
Peripheral TCL, not otherwise specified	6 (17%)
Unspecified TCL	5 (14%)
Angioimmunoblastic TCL	3 (9%)
Enteropathy-associated TCL	3 (9%)
Cutaneous TCL	2 (6%)
Mycosis fungoides	2 (6%)
T-cell prolymphocytic leukemia	2 (6%)
Cytotoxic chemotherapy treatment (first-line)	(35/35)
CHOP/CHOEP	22 (63%)
VACOP-B	4 (11%)
ABVD	1 (3%)
Other	1 (3%)
None	7 (20%)

a Nodal localization: malignancy in lymph nodes only.

b Extranodal localization, other: pleura, lung, meninges, bone marrow, testis.

ABVD = adriamycin (doxorubicin), bleomycin, vinblastine, dacarbazine; CHOEP = addition of etoposide to the standard CHOP-regimen; CHOP = cyclophosphamide, hydroxydaunorubicin, oncovin (vincristine), prednisone or prednisolone; ECOG = Eastern Cooperative Oncology Group; IPI = International Prognostic Index; LDH = lactate dehydrogenase; TCL = T-cell lymphoma; VACOP-B = VP-16 (etoposide), adriamycin (doxorubicin), cyclophosphamide, oncovin (vincristine), prednisone, bleomycin; WHO = World Health Organization.

#### Expression of FoxP3 in TCL

**Figure 1.**
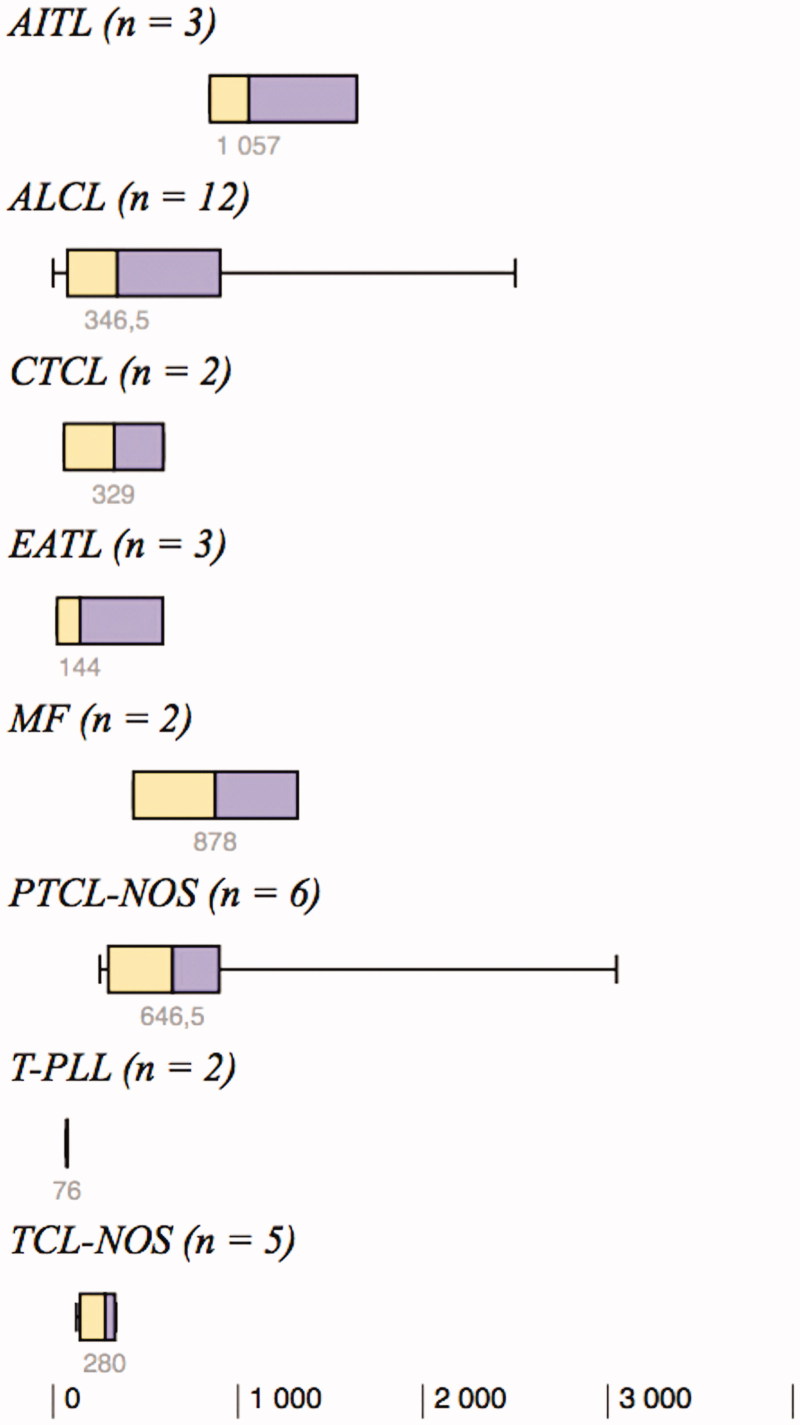
Degree of intratumoral expression of FoxP3^+^ cells/mm^2^ in the eight different subtypes of T-cell lymphoma. AITL: angioimmunoblastic T-cell lymphoma; ALCL: anaplastic large cell lymphoma; CTCL: unspecified cutaneous T-cell lymphoma; EATL: enteropathy-associated T-cell lymphoma; MF: mycosis fungoides; PTCL-NOS: peripheral T-cell lymphoma, not otherwise specified; T-PLL: T-cell prolymphocytic leukemia; TCL-NOS: unspecified T-cell lymphoma.

All 35 tissue samples expressed FoxP3 to some degree. Overall, the median expression of FoxP3 was 342 positive cells/mm^2^ (mean 610 positive cells/mm^2^), and the range was large (1–3047 positive cells/mm^2^). The degree of intratumoral expression of FoxP3 tended to vary between the different subtypes of TCL (Figure 1). The highest frequency of FoxP3^+^ cells was detected in AITL (*n* = 3, median 1057 and range 849–1642 positive cells/mm^2^) compared with all other subtypes, although this difference did not reach statistical significance (*P* = 0.053). T-PLL (*n* = 2) tended to have the lowest frequency of FoxP3^+^ cells/mm^2^ (*P* = 0.14).

#### Survival

**Figure 2.**
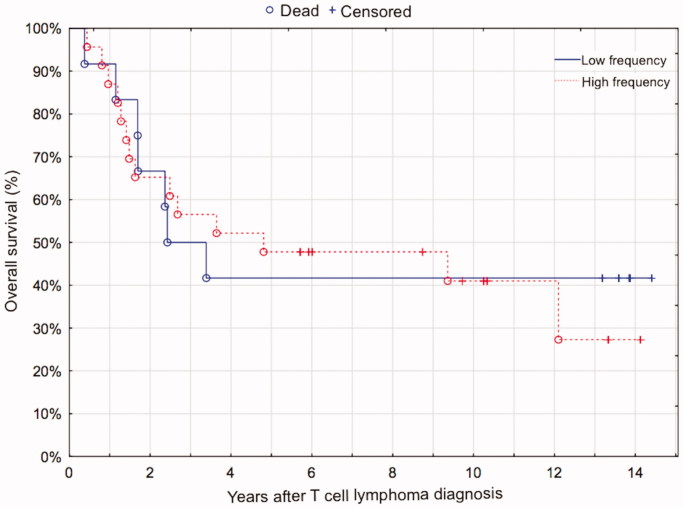
The frequency of intratumoral FoxP3^+^ cells (more or less than 200 FoxP3^+^ cells/mm^2^) in T-cell lymphomas had no impact on overall survival.

**Figure 3.**
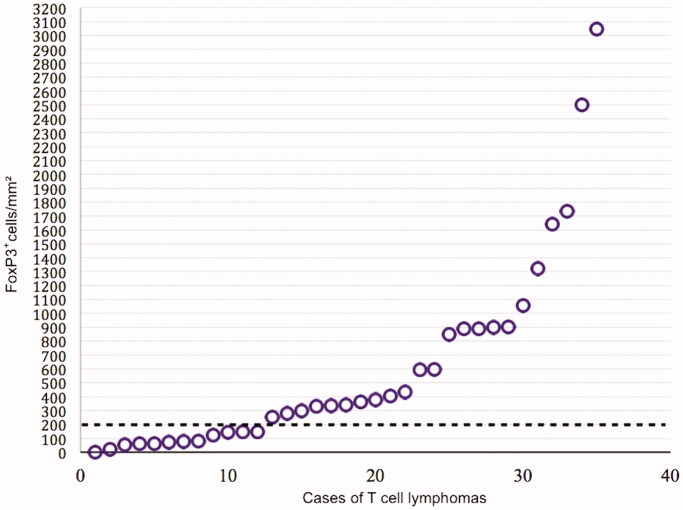
Degree of intratumoral expression of FoxP3 in the 35 biopsies, with cutoff level set arbitrarily at 200 FoxP3^+^ cells/mm^2^.

At the end of follow-up 21 patients had died, 18 of whom due to lymphoma. The median follow-up time for the 14 surviving patients was 11.8 years (range 5.7–14.4 years). One- and five-year overall survival were 89% and 46%, and one- and five-year relapse-free survival were 72% and 44% for all patients. There was no difference in OS between cases with high or low expression of FoxP3, neither when analyzed as a dichotomous variable (> or < 200 FoxP3^+^ cells per mm^2^, *P* = 0.84, Figure 2), nor as a continuous variable (*P* = 0.63). This result did not change when the cases of MF, which have a better prognosis than the rest of the TCLs, were omitted from the survival analysis (log-rank test, *P* = 0.55; Cox proportional hazards, *P* = 0.76). Considering that no difference in OS was detected when analyzing FoxP3 as a continuous variable, it is of minor consequence that the cutoff was set arbitrarily based on visual assessment of a scatter chart (Figure 3) when FoxP3 was used as a dichotomous variable.

## Discussion

In this study we found that all TCLs expressed FoxP3 to some degree but that the frequency varied considerably between different subtypes. There was no association between the number of intratumoral Tregs and OS.

Many studies have shown a correlation between a high density of Tregs in tumor tissue and a worse prognosis in different malignancies (11–13), but at the same time several studies have shown the opposite (14–17). The negative impact of Tregs on outcome in cancers may be explained as one of several immune evasion strategies by tumors, where the tumors escape the immune system by suppressing antitumoral effector cells (11,22–24). In the malignancies where Tregs instead are associated with a favorable outcome, a possible explanation could be that Tregs, due to their anti-inflammatory capacity, reduce tissue damage and restrict inflammation-related carcinogenesis (1,25,26).

In TCL, the function of Tregs seems particularly complex, partly because the malignant cells in these tumors are lymphoid and thus potentially could be suppressed by Tregs, and partly because some studies have suggested that the malignant cells could in fact be Treg-derived (19,27–29).

Berger et al. (29) suggested that CTCLs are tumors of malignant Tregs, based on findings that CTCL-cells could adopt Treg phenotype and function after stimulation by apoptotic material *in vitro*. Gjerdrum et al. (16) showed that only a small minority of CTCL cases showed expression of FoxP3 in the malignant cells while all contained FoxP3^+^ cells in the tumor microenvironment to some degree. Of note, higher numbers of intratumoral Tregs were associated with improved survival in these TCLs. There were only two MF and two CTCL unspecified in our case series, and we have not used the same method for evaluation; this complicates comparisons between the two studies, but the frequencies of Tregs seem similar.

Regarding ALCL, PTCL-NOS, and AITL, we found higher frequencies of FoxP3^+^ cells than previous studies (15,18,27). The largest subgroup in our case series was ALCL (*n* = 12), where we found a mean of 621 FoxP3^+^ cells/mm^2^ as compared with 15 FoxP3^+^ cells/mm^2^ in the four cases of ALCL in the study by Tzankov et al. (15). Bonzheim et al. (27) detected FoxP3^+^ cells in four of six ALK^+^ and in one of 14 ALK^-^ ALCL, but the specific number of positive cells was not reported. This is in sharp contrast to our case series where the majority of ALCL were ALK^-^ (seven of nine with known ALK status) and despite this were FoxP3^+^. However, the limited number of cases prevents a meaningful comparison between ALK^+^ and ALK^-^ ALCL. In PTCL-NOS we found a mean of 965 FoxP3^+^ cells/mm^2^ compared with 34 FoxP3^+^ cells/mm^2^ in the 27 cases in the study by Tzankov et al. (15), whereas Bonzheim et al. (27) only detected expression of FoxP3 in one of 14 cases of PTCL-NOS, and that was in the malignant population. Regarding AITL, we detected a mean of 1183 FoxP3^+^ cells/mm^2^ in three cases as compared with a mean of 61 FoxP3^+^ cells/mm^2^ in 23 cases by Tzankov et al. (15) and mean of 90 FoxP3^+^ cells/high-power field (HPF; 400×) in 30 cases by Bruneau et al. (18). Bonzheim et al. (27) found no FoxP3^+^ tumor cells and only a few FoxP3^+^ cells in the reactive infiltrate in 23 cases of AITL.

The differences in expression of FoxP3 can in part be explained by the use of different antibodies for the detection of FoxP3. Tzankov et al. (15) used the mouse monoclonal antibody 22510 (Abcam), Bonzheim et al. (27) used a rabbit polyclonal antibody from Abcam (clone number not specified), whereas Bruneau et al. (18) and Gjerdrum et al. (16) used the same clone as we did, namely the mouse monoclonal 236A/E7 (Abcam). Clone 236A/E7 has been reported to be predictive of improved outcome in follicular lymphoma (30), Hodgkin lymphoma (31), and CTCL (16). In our case series there was no correlation between expression of FoxP3^+^ and outcome. An association between high numbers of intratumoral Tregs and superior survival has been reported for some types of TCLs, e.g. CTCL (16) and ENKTL (17), but not for the largest subgroups in our case series, ALCL and PTCL-NOS, to the best of our knowledge.

In a previous study with the use of the same antibody as in the present study, we found that 13 cases of T-cell posttransplant lymphoproliferative disorder occurring after solid organ transplantation were *completely* negative for expression of FoxP3 and in one case FoxP3^+^ cells were detected at a low density (10 positive cells/mm^2^) (20). This finding is in contrast with the observation in the present study where all TCL expressed FoxP3 to some degree. A plausible explanation for this striking difference is the influence of the immunosuppressive drugs in solid organ transplant recipients.

The limitations of this study include the small subgroups of TCL subtypes, which hinders comparisons between groups. Further, the FoxP3^+^ cells in the biopsies have not been defined as belonging to either the neoplastic or reactive cell population within the tumors. The frequency of intratumoral Tregs is based on the expression of FoxP3, which has limitations but still is considered the most reliable biomarker for Tregs (10). Strengths include that the biopsies were collected prospectively on a national level and re-evaluated by experienced hematopathologists. Furthermore, 15 arbitrarily chosen HPFs were manually counted which is more than the standard five HPFs.

In conclusion, in this study we found distinctly higher levels of expression of FoxP3 in AITL, PTCL-NOS, and ALCL when compared with previous studies of these lymphomas in immunocompetent individuals. The finding that all of the TCLs showed expression of FoxP3 to some degree is interesting when compared with the results of our previous study of Tregs in posttransplant lymphoproliferative disorder, where all but one of the T-cell posttransplant lymphoproliferative disorders were entirely lacking expression of FoxP3. Thus, the role of Tregs seems to differ between TCLs in immunocompetent and immunosuppressed hosts. In this heterogeneous group of TCL, there was no correlation between the density of intratumoral FoxP3^+^ cells in biopsies of TCL and OS.
